# The Story of the Insane from Year to Year

**Published:** 1905-02-11

**Authors:** 


### THE STORY OF THE INSANE FROM YEAR TO YEAR.

Seventeenth Annual Series.

### THE ROYAL ASYLUM, PERTH.

In this asylum the number was at the beginning of the year 132, and at the end of the year the same number was in residence. There were 31 first admissions, 6 readmissions, 16 recoveries, 8 discharged relieved, 4 not improved, and 9 died. The average number resident during the year was 131, and the total number under treatment was 169. The recoveries were 43·24 per cent. of the admissions, and the deaths were 6·84 of the average number resident. Three of the deaths during the year were caused by cerebral diseases, 3 by pneumonia, and 1 by consumption. The admissions were considerably fewer than in the previous year, and Dr. Urquhart seems inclined to think that the cool summer of 1903 had something to do with this diminution of the admission rate; but he adds that "it would be rash to draw definite conclusions from the limited statistics of the institution," and he reminds us that depression in trade will influence the statistics of insanity, as "it has been repeatedly shown that prosperity and high wages mean much drunkenness and much insanity." Of the year's admissions 22 were hereditarily disposed to insanity, 5 belonged to neurotic families, and 3 cases were of alcoholic origin. Of those remaining in residence at the end of the year 14 were suicidal, 7 were epileptic, and 5 were general paralytics. As regards prospects of cure, Dr. Urquhart only places 12 of the whole of the patients in the asylum as at all likely to recover. Of the 169 cases under treatment during the year not fewer than 51 were allowed to go out unattended on parole, and only in one case was the parole broken. The patients on the register paid an aggregate sum of £12,950, being about £94 per annum per patient. The ordinary minimum rate for board is £84, but we are pleased to note that 34 patients were admitted at sums varying from £26 to £52.

